# Policy Design of Multi-Year Crop Insurance Contracts with Partial Payments

**DOI:** 10.1371/journal.pone.0145384

**Published:** 2015-12-22

**Authors:** Ying-Erh Chen, Barry K. Goodwin

**Affiliations:** 1 Department of Insurance, Tamkang University, Tamsui Dist., New Taipei City, Taiwan; 2 Departments of Agricultural and Resource Economics and Economics, North Carolina State University, Raleigh, North Carolina, United States of America; Iowa State University, UNITED STATES

## Abstract

Current crop insurance is designed to mitigate monetary fluctuations resulting from yield losses for a specific year. However, yield realization tendency can vary from year to year and may depend on the correlation of yield realizations across years. When the current single-year Yield Protection (YP) and Area Risk Protection Insurance (ARPI) contracts are extended to multiple periods, actuarially fair premium rate is expected to decrease as poor yield realizations in a year can be offset by another year’s better yield realizations. In this study, we first use simulations to demonstrate how significant premium savings are possible when coverage is based on the sum of yields across years rather than on a year-by-year basis. We then describe the design of a multi-year framework of crop insurance and model the insurance using a copula approach. Insurance terms are extended to more than a year and the premium, liability, and indemnity are determined by a multi-year term. Moreover, partial payment is provided at the end of each term to offset the possibility of significant loss in a single term. County-level data obtained from the U.S. Department of Agriculture are used to demonstrate the implementations of the proposed multi-year crop insurance. The proposed multi-year plan would benefit farmers by offering insurance guarantees across years for significantly lower costs.

## Introduction

Agricultural production suffers potential risks because of instabilities in yield and price. These instabilities can result from various unpredictable factors, including natural disasters such as fire, drought, floods, and pest infestations. Yield volatility causes price movements and income instability for farmers. The U.S. Federal Crop Insurance Program provides various types of insurance to help protect farmers from production, price, and income risks.

Some insurance is based at the farm-level, such as farm-level yield insurance (Yield Protection Policy) and farm-level revenue insurance (Revenue Protection Policies and Revenue Protection with Harvest Price Exclusion Policies). The Yield Protection (YP) policy protects farmers against individual yield losses, and the production guarantee is based on the actual production history (APH) yield, which is an average of four to ten years of realized yields for the insured unit. Insured farmers are qualified to obtain indemnities when actual yields fall short of insured yields. The indemnity is calculated as the difference between the production guarantee and actual yields multiplied by the price election. Revenue Protection (RP) Policy offers a revenue guarantee based on the APH yield and the projected and harvest prices, depending on which is greater. RP with the Harvest Price Exclusion (RPHPE) Policy is similar to RP. The difference is that revenue guarantee is determined by the projected price only and therefore excludes the possibility of benefiting from higher harvest prices.

Other types of crop insurance are based on an index or aggregated yields at an area level such as county-level Area Risk Protection Insurance (ARPI). ARPI provides Area Yield Protection (AYP), Area Revenue Protection (ARP), and Area Revenue Protection with Harvest Price Exclusion (ARPHPE). AYP is similar to the YP policy, except that AYP uses county yields instead of unit yields for the indemnity calculation. ARP offers protection against loss of revenue resulting from production loss, price decline, or both and includes upside harvest price protection. ARP is similar to RP, except that ARP uses county-level revenue instead of unit revenue for the indemnity calculation. ARPHPE is similar to ARP but it excludes upside harvest price protection. ARPHPE is similar to RPHPR, except that ARPHE uses county-level revenue instead of unit revenue for the indemnity calculation.

Currently, YP and ARPI are designed to mitigate monetary fluctuations resulting from yield losses for a specific year. However, yield realizations (or the yield realization tendency) can vary from year to year, and actuarially fair premium rate (AFPR), which is the expected loss divided by liability, may depend on the correlation of yield realizations across years. Many farm programs have as their stated intention the desire of policymakers to stabilize farmers’ incomes across years. If poor yield realizations in a year can be offset by another year’s better yield realizations, then AFPR is expected to decrease when the current single-year YP and ARPI contracts are extended to multiple periods.

Extending insurance terms to multiple periods has been considered for the design of insurance policies. Gardner and Kramer [1] showed that for full-coverage insurance contracts with infinitely long lives, moral hazard can be eliminated if at the time when cheating is detected, the insurer refuses to cover any future losses. Chambers [2] pointed out that moral hazard can reappear at the end of a finite multi-year contract, because the insurer has no future recourse against the insured farmer and, therefore, has no credible threat to enforce honest behavior. However, if penalties for shirking are sufficiently high during a multi-year contract, moral hazard is likely mitigated [2,3]. Kleindorfer et al. [4] investigated the demand and supply of single- and multi-year property insurance in the presence of risks such as hurricanes and earthquakes, and concluded that single- and multi-year policies should be both provided as consumers have different degrees of risk aversion. Maynard and Ranger [5] showed that a longer contract is more beneficial to incentivize risk reduction. Osipenko et al. [6] adopted a discrete choice model to show that there is a demand for multi-year crop insurance and that single- and multi-year cop insurance plans can co-exist. As these studies suggest although there is a viable market for multi-year insurance, the specific design of multi-year crop insurance has not yet been fully investigated.

Here, we propose multi-year YP and ARPI. Insurance terms are extended to more than a year, and the premium, liability, and indemnity are determined by a multi-year term. Moreover, partial payment is provided at the end of each term to offset the possibility of a significant loss in a single term for farmers. We used computer simulations to demonstrate that when annual yields across years are not perfectly correlated across years, significant premium savings are possible when coverage is based on yields that are summed across years rather than on a year-by-year basis. We then use real county-level yield data in the U.S. to show that most of the yield data across years are not significantly correlated. Finally, we use yield data from Adair County, Iowa to demonstrate the implementation of the proposed two- and three-year crop insurance plans.

## Materials and Methods

### A Novel Design of Multi-year Crop Insurance Plans

We use YP to demonstrate how the plan would be extended to a multi-year framework. For a multi-year YP, farmers would choose a coverage level, identical to the current YP. However, two major differences exist between a multi-year YP and the current YP. The first major difference is that insurance would be extended from a single-term to multiple periods of time. The guaranteed yield would be calculated for each period in a way that is similar to the current YP and the payment in each period is referred to as the *partial payment*. The second main difference is that the guaranteed yield for the multi-year plan would be based on sums of the actual yield over a period of multiple years. Payments would be made in the last harvest period when the sums of the actual yield over the specified period of time falls below the guaranteed yield. Payments made based on the sum of actual yields is referred to as *total indemnity*. Let us assume that there is a two-year YP and there is a decrease in the yield in the first year, which is offset by an increase in the yield in the second year (or vice versa). In this case, farmers would pay a lower premium cost and the government would maintain the subsidy rate.

To help farmers prepare for a possible yield loss in either one of the insured years in this example of two-year YP, insured farmers would obtain a partial payment in a certain insured year if the actual yield during that year falls below a certain level. Therefore, the two-year YP would provide a combination of both single and multi-year coverages. Let us also assume that the two-year YP guarantees 50% of the yield in each of the two years for partial payment and guarantees 85% of the sum of the yields over the two years. If a farmer’s realized yield in either one of the two years is below the guaranteed yield (i.e., 50% of the predicted yield), the farmer will obtain a partial payment, calculated based on the difference between the guaranteed and realized yields for the year and price election. In the second year, the farmer will receive indemnities if the realized yield over the two years is less than the guaranteed yield (i.e., less than 85% of the sum of the yields over the two years).

Since farmers may be dependent on loans, the partial payment will be used to cover their short-term debt. Therefore, the partial payment in each insured year assures that the yield loss will not bankrupt farmers and instead will help farmers repay their operation loans. An important question is to determine the coverage level for the partial payment. The variable costs of production from extension service available in many states in the U.S. and the predicted price from the Chicago Mercantile Exchange & Chicago Board of Trade (CME Group Inc.) can be used to determine the coverage level for the partial payment. The single-year coverage level (*γ*) of partial payment is calculated as:

$$\gamma =\frac{\text{estimated variable cost}}{\text{predicted price}}$$ (1)

The single-year coverage level (*γ*) calculated based on the variable cost of production and futures price is considered as the lowest single-year coverage level set by policy makers when implementing a multi-year crop insurance contract because the partial payment would cover the basic operation loans for farmers. Farmer could select the single-year coverage level above the single-year coverage level set by policy makers. However, selection of the single-year coverage-level needs to be lower than the two-year coverage level. Otherwise, it will contradict the goal of the partial payment and the design of the multi-year crop insurance to benefit farmers through sum of yields across years.

We will now formally introduce how the partial payment and indemnity will be made with the following equations in a two-year YP. For simplicity, we assume that the price election is $1, which means that the partial payment and indemnity can be directly calculated as the difference between the realized yield and guaranteed yield. We first introduce some notations as follows:

${Pay}_{i}(y_{i}^{A},\gamma )$: partial payment made in year *i*, *i* = 1, 2;

$y_{i}^{A}$: the actual yield in year *i*, *i* = 1, 2;

*γ*: the single-year coverage level for the partial payment determined by farmers and 0<*γ*<1;

*β*: the multi-year coverage level for the indemnity decided by farmers and 0<*β*<1;

*μ*: the expected yield and *γμ* is the threshold of the partial payment.

The partial payment can be calculated as:

$$Pay_{i}(y_{i}^{A},\gamma )=\text{max}(\gamma \mu -y_{i}^{A},0)$$ (2)

The partial payment will be made in year *i* if $y_{i}^{A}<\gamma \mu$. A total indemnity will be made in the second year if the following condition is met:

$$\text{max}(y_{1}^{A},\gamma \mu )+y_{2}^{A}<2\beta \mu$$ (3)

Therefore, the partial payment and total indemnity made in the second year could be expressed as:

$$\begin{array}{l} Pay_{2}(y_{2}^{A},\gamma )+TI(y_{1}^{A},y_{2}^{A}|\gamma ,\beta )\\ =\text{max}(\gamma \mu -y_{2}^{A},0)+\text{max}\{2\beta \mu -\text{max}(y_{1}^{A},\gamma \mu )-\text{max}(y_{2}^{A},\gamma \mu ),0\} \end{array}$$ (4)

AFPR of the two-year YP is given by the ratio of the expected loss to total liability, given as:

AFPR=prob(y1<γμ)max(γμ−y1A,0)+prob(y2<γμ)max(γμ−y2A,0)+prob(y1+y2<2βμ)max(2βμ−max(y1A,γμ)−max(y2A,γμ),0}2βμ=prob(y1<γμ)[γμ−E(y1|y1<γμ)]+prob(y2<γμ)[γμ−E(y2|y2<γμ)]+prob(y1+y2<2βμ)[2βμ−(max(y1A,γμ)−max(y2A,γμ))]2βμ (5)

The numerator of Eq 5 is the expected loss, which consists of three parts. The first two parts are the products of the probability that a loss is realized times the expected loss given that the loss occurs for the two insured years. Here, a loss occurs when a single-year yield is less than γ×100% of the predicted yield in an insured year. The third part is the product of the probability that a loss is realized times the expected loss given that the loss occurs at the end of the second insured year. Here, a loss occurs when the sum of the yields across the two years is less than β×100% of sum of the predicted yields of the two years. The actuarially fair premium per acre is the product of guaranteed yield (2*βμ*), price election and AFPR. AFPR of a multi-year insurance contract is a combination of both the single- and multi-year coverages. To derive a valid measure of AFPR of a multi-year insurance contract, the correlation structure of the yields between each individual insured year needs to be modeled to obtain a joint distribution of yields for individual years.

### Graphical Illustration of the Payment Structure in a Two-Year YP

**Fig 1:**
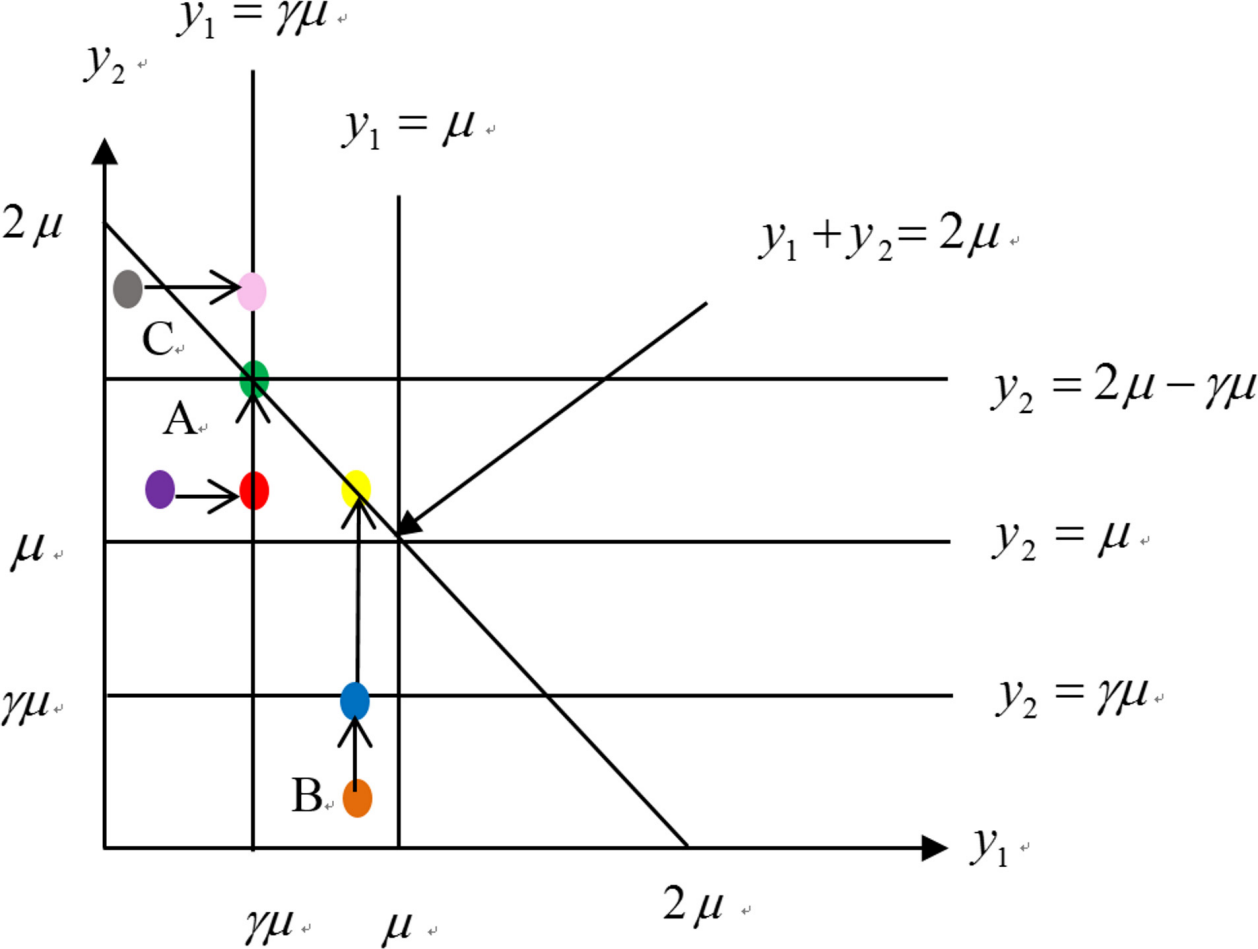
Illustration of the payment structures for a two-year YP.

We illustrate the payment structure, including the partial payment and total indemnity of a two-year YP, in Fig 1. We assume that *β* is 100%, which implies that insured farmers will obtain total indemnity in the second insured year when the sum of the realized yield in the first and second years falls below 2*μ*. Because the partial payment and indemnity can be directly calculated as the difference between the realized yield and guaranteed yield based on the assumption that the price election is $1, the realized yield and payments can be expressed in the same figure. In Fig 1, the horizontal and vertical axes are in dollar amounts (same as for yield) in the first and second years, respectively. We assume that $y_{1}^{A}$ and $y_{2}^{A}$ both fall inside area “A” and that the coordinate for ($y_{1}^{A}$, $y_{2}^{A}$) is (*a*, *b*), shown as a purple circle, where *a* < *γμ* and *b* > *γμ*. In the first year, since $y_{1}^{A}$ < *γμ*, the partial payment will be made in the first year according to Eq 2. The value of the partial payment is the distance between *a* and *γμ*, and we shift the coordinate ($y_{1}^{A}$, $y_{2}^{A}$) horizontally from (*a*, *b*) to (*γμ*, *b*) shown as a red circle. The move from the purple circle to the red circle in Fig 1 shows the amount of the partial payment that the farmer will obtain in the first year. This can also help us in the next step to decide if the partial payment and indemnity need to be made in the second year.

In the second year, since the realized yield is higher than *γμ*, shown as the red circle, the partial payment will not be made according to Eq 2. Because the farmer has obtained the partial payment in the first year, the yield in the first year is considered to be *γμ* instead of *a* when we decide if the farmer should obtain an indemnity in the second year. As a result, in the second year, because $\gamma \mu +y_{2}^{A}<2\mu$, the indemnity will be made according to Eq 3. The value of the indemnity paid to the farmer can be shown as the distance from *b* to 2*μ—γμ* (i.e., the distance between the red and green circles).

Second, we consider another situation where both the partial payment and total indemnity are made in the second year. We assume that $y_{1}^{A}$ and $y_{2}^{A}$ fall inside area “B” and that the coordinate for ($y_{1}^{A}$, $y_{2}^{A}$) is (*c*, *d*), shown as an orange circle in Fig 1, where *c* > *γμ* and *d* < *γμ*. In the first year, the partial payment will not be made since $y_{1}^{A}$ > *γμ*. In the second year, the partial payment will be made since $y_{2}^{A}$ < *γμ*. The value of the partial payment paid to farmers in the second year is the distance between *d* and *γμ*. In the second year, we shift ($y_{1}^{A}$, $y_{2}^{A}$) vertically from (*c*, *d*) to (*c*, *γμ*) shown as a blue circle. Since (*c*, *γμ*) falls inside the line $y_{1}^{A}+y_{2}^{A}=2\mu$, which means that the sum of the yields across two years is lower than the guaranteed yield, the total indemnity will be made in the second year. To calculate the value of total indemnity paid to farmers, we shift (*c*, *γμ*) vertically to (*c*, *2μ-c*), which lies on the line $y_{1}^{A}+y_{2}^{A}=2\mu$ and is shown as a yellow circle. The value of the total indemnity paid to farmers in the second year is the distance from *γμ* to 2*μ*-*c*.

Finally, we consider the situation that the partial payment is made in the first year. We assume that $y_{1}^{A}$ and $y_{2}^{A}$ fall inside area “C” and that the coordinate for ($y_{1}^{A}$, $y_{2}^{A}$) is (*e*, *f*), shown as a gray circle in Fig 1, where *c* < *γμ* and *d* > *γμ*. In the first year, the partial payment will be made since $y_{1}^{A}$ < *γμ*. The value of the partial payment is the distance between *e* and *γμ*, and we shift the coordinate ($y_{1}^{A}$, $y_{2}^{A}$) horizontally from (*e*, *f*) to (*γμ*, *f*) shown as a pink circle. The move from the gray circle to the pink circle in Fig 1 shows the amount of the partial payment that the farmer will obtain in the first year. In the second year, the partial payment will not be made since $y_{2}^{A}$ > *γμ*. Since (*γμ*,*f*) falls outside the line $y_{1}^{A}+y_{2}^{A}=2\mu$, which means that the sum of the yields across two years is higher than the guaranteed yield, the total indemnity will not be made in the second year. In this situation, our two-year YP assumed that farmers can keep the partial payment obtained in the first year even when the sum of yield is higher than the guaranteed yield in the second year.

### Simulations for Corn in Adair County, Iowa

We use simulations to investigate the relationship between the AFPR and the correlation of yields across years for two-year and three-year YPs. We use county-level data for Adair County in Iowa, obtained from the National Agricultural Statistical Service (NASS) of the U.S. Department of Agriculture (USDA). Detrend and normalization for the data are described in detail in the section of Empirical Analysis below. The Empirical Analysis Section also describes the goodness-of-fit tests which find that a beta distribution is appropriate for the yields. Thus, we simulated yields using beta distributions. In the simulations, correlation coefficients of yield distributions between the first and second years vary between −1 and 1. The pairwise correlation coefficients of the yield distributions among the first, second, and third years are assumed to be the same and are between −0.5 and 1. We simulate two scenarios. In the first scenario, the farmer selects a 54% single-year coverage level and a 70% two-year coverage level. In the second scenario, the farmer selects a 54% single-year coverage level and a 80% two-year coverage level. The single-year coverage level (i.e., 54%) is calculated by the ratio of the variable cost of Iowa corn divided by corn futures. Production costs of Iowa corn are obtained from the Iowa State Extension Services and futures are obtained from CME Group Inc. In this simulation, the estimated variable cost of production per bushel is 2.75 U.S. dollars and the corn futures was 5.11 U.S. dollars per bushel in December of 2014.

Table 1 shows the simulation results for the first and second scenarios. As seen in Table 1, when the yields between two years are perfectly positively correlated, AFPR of a two-year YP is at its maximum and is similar to a single-year YP. This result also holds true for the three-year YP. When the correlation of the yields between years decreases, AFPRs of the two- and three-YPs also decrease. The simulation results demonstrate that a multi-year YP could have a lower AFPR than the current single-year YP. More specifically, AFPR of a two-year YP could be lower than that of a single-year YP; AFPR of a three-year YP could also be lower than that of a single-year YP. Therefore, the superiority of a multi-year YP is that it provides farmers an opportunity to have lower AFPR by combining yields across two or three years. The simulation results are also as expected in the statistical sense because lower correlation between two random variables (i.e., yields of years 1 and 2) decreases the variance of their sum.

**Table 1:** Actuarially fair premium rates (AFPR) for two-and three-year YPs.

	Scenario 1		Scenario 2	
Correlation of yield across years	AFPR for two-year YP	AFPR for three-year YP	AFPR for two-year YP	AFPR for three-year YP
-1.0	0.0014	N/A	0.0012	N/A
-0.9	0.0014	N/A	0.0012	N/A
-0.8	0.0014	N/A	0.0012	N/A
-0.7	0.0014	N/A	0.0014	N/A
-0.6	0.0013	N/A	0.0017	N/A
-0.5	0.0014	0.0014	0.0022	0.0012
-0.4	0.0015	0.0013	0.0030	0.0012
-0.3	0.0016	0.0014	0.0038	0.0013
-0.2	0.0018	0.0014	0.0050	0.0017
-0.1	0.0023	0.0015	0.0062	0.0024
0.0	0.0025	0.0017	0.0075	0.0037
0.1	0.0030	0.0018	0.0089	0.0050
0.2	0.0034	0.0022	0.0103	0.0066
0.3	0.0039	0.0027	0.0119	0.0085
0.4	0.0046	0.0035	0.0135	0.0103
0.5	0.0055	0.0041	0.0152	0.0123
0.6	0.0063	0.0050	0.0166	0.0147
0.7	0.0072	0.0062	0.0187	0.0166
0.8	0.0081	0.0075	0.0199	0.0190
0.9	0.0094	0.0087	0.0217	0.0215
1.0	0.0102	0.0104	0.0232	0.0237

Note: AFPRs for a single year plan are 0.0102 and 0.0232 for Scenarios 1 and 2, respectively, which are similar to the rates for multi-year YPs when the correlation coefficient is 1.

Note that when the single-year coverage level equals the two-year coverage level, purchasing a two-year YP is equal to purchasing two single-year YPs for farmers. To further examine the relationship between the single-year and two-year coverage levels, we estimated the set of single-year and two-year coverage levels given the same AFPRs under three scenarios (i.e., AFPRs are 0.015, 0.007, and 0.002 when correlations of yields across years are 0.5, 0, and -0.5, respectively). We assumed that single-year coverage level was not higher than the two-year coverage level, as the design of partial payment is to help farmers to repay their basic operation loans. The results are shown in Table 2. As seen in Table 2, when the single-year coverage level decreased, the two-year coverage level increased in all three scenarios. The results demonstrated the tradeoff of selecting between the single- and two-year coverage levels.

**Table 2:** The set of two-year coverage level (*β*) and one-year coverage level (*γ*) given the same AFPR for different levels of correlation for yields across years.

Scenario 1		Scenario 2		Scenario 3	
Correlation = 0.5, AFPR = 0.015		Correlation = 0, AFPR = 0.007		Correlation = -0.5, AFPR = 0.002	
*β*	*γ*	*β*	*γ*	*β*	*γ*
0.748	0.746	0.668	0.662	0.566	0.562
0.759	0.740	0.720	0.657	0.599	0.559
0.784	0.721	0.734	0.655	0.608	0.558
0.789	0.706	0.747	0.655	0.617	0.555
0.794	0.684	0.750	0.647	0.623	0.558
0.797	0.634	0.767	0.636	0.635	0.558
0.800	0.597	0.771	0.627	0.786	0.549
0.802	0.576	0.782	0.621	0.797	0.540
0.807	0.533	0.765	0.602	0.804	0.531
0.808	0.505	0.808	0.513	0.810	0.507

### Empirical Analysis

The simulation results show that the correlation of yields across years affects AFPR in the multi-year YP. Therefore, we use county-level data for corn in several important producing U.S. counties and states to investigate the level of the correlation of yields across years using real data.

The county-level data include county corn yield data in both insured and uninsured farms in Iowa, Illinois, Ohio, and Indiana where corn is the predominant crop from 1933 to 2012. The data are also from the NASS of the USDA. The average county yields of corn over 80 years in Iowa, Illinois, Ohio, and Indiana were calculated, and the top ten production counties in these four states were chosen for our analysis.

#### Yield Trend Estimation

Crop yields have trended upward because of improved production techniques. Thus, crop yields over different periods of time cannot be combined. Any inference about yield risk in the period being evaluated for insurance products will be biased with a failure to detrend crop yields [7–9]. Thus, we need to remove the time trend for the yield data collected over different years. First, we fit the time trend model for crop yields over years. The relationship between the crop yield *y*_*t*_ and time can be represented as

$$y_{t}=X_{t}\beta +e_{t}$$ (6)

where *X*_*t*_ represents the linear or nonlinear function of time.

Our regression analysis suggest that corn yields of county data grew quadratically (i.e., the null hypothesis of *β*_*2*_ = 0 was rejected) (data not shown) and thus the following equation is used to remove the time trend:

$$y_{t}=\beta_{0}+\beta_{1}t+\beta_{2}t^{2}+e_{t}$$ (7)

After we fit the model for the crop yield over time, we obtain trend-predicted yield data (${\hat{y}}_{t}$) and the deviation from the trend (*e*_*t*_). Since a lot of empirical studies support the idea that deviations from time tend to be proportional to crop yield, we use the following equation to normalize crop yields over time [10]:

$$\tilde{y_{t}}=\hat{y_{T}}\left(1+\frac{e_{t}}{\hat{y_{t}}}\right)$$ (8)

where $\hat{y_{T}}$ is detrended yield data of the last observation and $\tilde{y_{t}}$ is normalized yield data over time.

#### Autocorrelation coefficient of yields across years

We use an autoregressive (AR) model to estimate the autocorrelation of yields across years. After detrending the normalized county yield data, autocorrelation coefficients of order 1 are estimated and the significance of the autocorrelations between *y*_*1*_ and *y*_*2*_, which are yields between the two years, are tested.

### A Demonstration of Two- and Three-Year YPs using Data from Adair County, Iowa

We use county-level data from Adair County, Iowa from the NASS of the USDA to demonstrate the implementation of two- and three-year YPs. We also use Eqs 6 and 8 to detrend and normalize the corn yield data. As AFPR for a multi-year insurance plan is calculated based on the joint distribution of yields in individual insured years, we need to model the joint distribution and estimate correlations of yields across years. The modeling of a joint distribution can become complicated when marginal distributions do not have a closed form expression. Recently, the copula model has become a popular approach to modeling joint distributions in agriculture [11–14]. A copula provides flexibility as a dependence function that binds marginal distributions together to express joint distribution without sacrificing the properties of marginal distributions. Because marginal distributions need to be specified for copula and normal and beta distributions have been commonly adopted to model yield distributions in the literature [15–20], we perform goodness-of-fit tests, including the Kolmogorov-Smirov, Cramer-von Mises, and Anderson-Darlilng tests for testing normality and the Chi-square test for testing normal and beta distributions using SAS software to select a proper distribution for the yield data.

We consider several copulas, including the Frank, Clayton, Normal, and Gumbel copulas. Parameters for the marginal distributions and the copula function are jointly estimated. As these four copulas have the same number of parameters in a two-year YP, we selected the best model based on their likelihood values. For a three-year YP where copulas could have a different number of parameters based on the correlation matrix structure, we selected the best model based on their Akaike information criterion (AIC) values. After a proper copula model was selected, correlated yield data were simulated based on the model. Then AFPRs were calculated on the basis of Eq 5 for the simulated data.

## Results

### Autocorrelation Coefficient of Yields across Years Based on County-Level Data

Autocorrelation coefficients of order 1 and p-values are shown in Table 3. Interestingly, different correlation patterns are observed among the four states. The results from west to east are: in Iowa, autocorrelation coefficients are all positive in the top ten production counties; in Illinois, autocorrelation coefficients are all positive, except in Champaign and De Witt counties; in Indiana, autocorrelation coefficients are all negative, except in Rush and Union counties; and in Ohio, autocorrelation coefficients are mostly negative, except in Fulton, Henry, Lucas, and Sandusky counties. However, correlations in these counties are not statistically significant, except for Kossuth County in Iowa which has a correlation of 0.303 with a p-value of 0.004 at the significance level of 0.01.

**Table 3:** Correlation results for the top 10 production counties in four states based on county-level NASS data.

Iowa		Illinois		Indiana		Ohio	
Cedar	0.015^1^ (0.894)^2^	Carroll	0.103 (0.351)	Benton	-0.102 (0.353)	Champaign	-0.078 (0.482)
Grundy	0.164 (0.137)	Champaign	-0.011 (0.922)	Carroll	-0.192 (0.071)	Clark	-0.174 (0.112)
Hamilton	0.177 (0.099)	De Kalb	0.065 (0.558)	Clinton	-0.213 (0.051)	Clinton	-0.148 (0.179)
Hardin	0.135 (0.223)	De Witt	0.000 (0.997)	Howard	-0.164 (0.134)	Fayette	-0.223 (0.039)
Humboldt	0.172 (0.114)	Logan	-0.094 (0.364)	Madison	-0.076 (0.487)	Fulton	0.171 (0.110)
Kossuth	**0.303 (0.004)**	Macon	0.058 (0.592)	Newton	-0.061 (0.583)	Greene	-0.102 (0.356)
Marshall	0.138 (0.212)	Moultrie	0.180 (0.097)	Rush	0.031 (0.765)	Henry	0.173 (0.098)
Scott	0.017 (0.876)	Piatt	0.043 (0.694)	Tipton	-0.172 (0.113)	Lucas	0.176 (0.105)
Webster	0.224 (0.036)	Sangamon	0.086 (0.436)	Union	0.084 (0.428)	Miami	-0.114 (0.293)
Wright	0.231 (0.030)	Warren	0.058 (0.592)	White	-0.098 (0.369)	Sandusky	0.160 (0.148)

^1^Correlation coefficient

^2^P-value for testing the significance of correlation

The autocorrelation coefficients of order 1 in Table 3 are generally small and thus the AFPR of a multi-year crop insurance contract is expected to be lower than the current single-year crop insurance contract. Besides, the results in Table 3 provide valuable information for policy makers about the selection of pilot areas and priority areas to implement the multi-year crop insurance contract. That is, counties with smaller correlations can be selected as pilot areas because the multi-year crop insurance contract would be more advantageous in terms of AFPR over single-year contracts in these counties.

### A Demonstration of Two- and Three-Year Insurance Contracts using Data from Adair County, Iowa

The parameter estimates for the detrended data, as well as the goodness-of-fit test results are shown in S1 Table. The goodness-of-fit tests are rejected at the 0.01 significance level for normal distribution (Cramer-von Mises, Anderson-Darliling, and Chi-Square tests), but not rejected for beta distribution. Thus, we specify beta distributions as the marginal distributions for the Adair corn yield and model their joint distribution in a copula. The Clayton copula has the highest likelihood value and the lowest AIC value among the four copulas in two- and three-year yield protection policies, respectively. Therefore, the Clayton copula is used to simulate the correlated yield data. The results for the copula estimation are shown in Table 4.

**Table 4:** Estimation results based on Clayton copula.

Two-Year ARPI		
Variable	Parameter estimate	Standard error
α_1_	8.4662	1.3299
β_1_	4.3822	0.6680
α_2_	8.3649	1.3228
β_2_	4.4373	0.6822
Spearman’s correlation(ρ)	0.2940	
Maximized log likelihood	103.4086	
Three-year ARPI		
α_1_	8.2587	1.3224
β_1_	4.2737	0.6627
α_2_	8.7414	1.3965
β_2_	4.5574	0.7083
α_3_	8.2128	1.3134
β_3_	4.3765	0.6790
ρ_1_	0.4149	
ρ_2_	0.4158	
ρ_3_	0.4145	
AIC = -288.9482		

We demonstrate how partial payments can help farmers recover from a yield loss in either one of the two years or yield losses in both years when insured farmers select 54% single-year coverage and 70% two-year coverage level (Table 5). The Spearman’s correlation coefficient between two years is 0.2940, which was estimated by the Clayton copula. Next, we simulate a joint yield distribution with two marginal beta distributions that have a 0.2940 correlation between years. The estimated AFPR based on the simulated model is $0.0053 for a two-year insurance contract. The expected yield is 160.99 bushels per acre, the realized yields are 80 and 165 bushels per acre in the first and second insured years, respectively, and the insured price is $2.50 per bushel. The average size of a corn farm in Iowa is 242 acres from the 2002 *Agricultural Census*. As realized yield in the first insured year (80 bushels/acre) is below the guaranteed yield (86.97 bushels/acre), farmers are eligible to obtain a partial payment of $4192.65 in the first year, which is shown in Table 5.

**Table 5:** Adair County in Iowa (Corn) for Two-and Three-Year YP (54% Single Year Coverage and 70% Two- Year Coverage).

	Two-Year Yield Protection Policy	Three-Year Yield Protection Policy
Spearman’s rank correlation	0.2940	0.4149, 0.4158, 0.4145
Parameter of Beta Distribution	(α_1_,β_1_) = (8.46,4.38) (α_2_,β_2_) = (8.36, 4.43)	(α_1_,β_1_) = (8.25,4.27) (α_2_,β_2_) = (8.74, 4.55) (α_3_,β_3_) = (8.21,4.37)
Actuarially fair premium rate	$0.0053	$0.0049
Expected yield	160.99^a^	160.99
Coverage level of guaranteed yield for two-(and three) year insurance contract	70%	70%
Guaranteed yield for two(three) years	225.38 (= 2*70%*160.99)	338.07 (= 3*70%*160.99)
Coverage level of partial payment	54%	54%
Guaranteed yield in each year	86.93 (= 54%*160.99)	86.93 (= 54%*160.99)
Realized yield in year 1 & 2 (&3)	80, 165	80, 165, 75
Partial payment	(1) $4192.65 (= 2.5*(86.93–80)*242) in year 1 (2) 0 in year 2 because realized yield in year 2(165 bushel/acre) is higher than guaranteed yield in each year (86.93 bushel/acre)	(1) $4192.65 (= 2.5*(86.93–80)*242) in year 1 (2) $7217.65 (= 2.5*(86.93–75)*242) in year 3
Indemnity	0^b^ (86.93+165>225.38)	0 (86.93+165+86.93>338.07)

^a^Yield is measured as bushel on a per acre basis.

^b^An indemnity will not be paid in the third insured year because the sum of realized yield is higher than the guaranteed yield.

Table 5 also includes an example of a three-year contract. The Spearman correlation coefficients estimated from the Clayton copula are 0.4149 between years one and two, 0.4158 between years one and three, and 0.4145 between years two and three. The estimated AFPR is 0.0049, based on the simulated model. The assumptions for the two-year YP also hold true for the three-year YP. These assumptions include the amount of the expected yield, the coverage levels of the indemnity and partial payment, the amount of the realized yield in the first and second years, the insured price, and the size of the farm. We assume that the realized yield in the third year is 75 bushels per acre. As the amount of yield in the first and third insured years is below the guaranteed yield (86.93 bushels/acre), farmers would obtain a partial payment equal to $4192.65 in the first year and $7217.654 in the third year. In the third insured year, the sum of yields is higher than the guaranteed yield. Therefore, farmers would not obtain indemnity.

Insured farmers may prefer higher two-year coverage levels and higher single-year coverage levels. Therefore, we also simulate a two-and three-year yield protection policy with a single-year coverage level of 60% and a two-year coverage level of 80% (shown in Table 6). If farmers select a higher two-year coverage level, AFPR increases from 0.005 to 0.016. As the amount of the realized yield in the first year is below the guaranteed yield (96.59 bushels/acre), farmers would obtain a partial payment of $10036.95. In addition, the sum of yields is below the guaranteed yield (386.37 bushels/acre) for the three-year YP and therefore farmers obtain an indemnity of $11966.9. We also demonstrate AFPRs for two- and three-year crop insurance using the same assumptions (i.e., the amount of expected yield, the coverage levels of indemnity and partial payment, and the amount of realized yield in the first and second years) that we made above with different coverage levels for total indemnity, namely, 85%, 90%, 95% and 100% (shown in Table 7). As expected, the AFPR increases with increasing coverage level and the AFPR for a three-year YP is lower than that for a two-year YP given a particular coverage level.

**Table 6:** Adair County in Iowa (Corn) for Two-and Three-Year YP (60% Single Year Coverage and 80% Two-Year Coverage).

	Two-Year Yield Protection Policy	Three-Year Yield Protection Policy
Actuarially fair premium rate	$0.016	$0.0154
Expected yield	160.99	160.99
Coverage level of guaranteed yield for two-(and three) year insurance contract	80%	80%
Guaranteed yield for two(three) years	257.58 (= 2*80%*160.99)	386.37 (= 3*80%*160.99)
Coverage level of partial payment	60%	60%
Guaranteed yield in each year	96.59 (60%*160.99)	96.59 (= 60%*160.99)
Realized yield in year 1 & 2 (&3)	80, 165	80,165, 105
Partial payment	$10036.95 = 2.5*(96.59–80)*242 in year 1	$10036.95 = 2.5*(96.59–80)*242 in year 1
Indemnity	0 (because 96.59+165>257.58)	$11966.9 = 2.5*[386.37-(96.59+165+105)]*242

**Table 7:** AFPR under Different Coverage Level of Indemnity (54% Single Year Coverage Level).

AFPR for Two-Year YP	AFPR for Three-Year YP	Coverage level for the Total Insured Year
$0.0259	$0.0248	85%
$0.0397	$0.0388	90%
$0.0588	$0.0574	95%
$0.0819	$0.0810	100%

## Discussion

We propose multi-year crop insurance plans, which are expected to have lower AFPRs than current single-year crop insurance plans. Based on our simulation results, the AFPRs were approximately 25% and 16% for the two- and three-year YPs, respectively, of the AFPR for the single-year YP when the correlation of yields between years was 0 in Scenario 1, while the AFPRs were approximately 32% and 16% for the two- and three-year YPs, respectively, of the AFPR for the single-year YP in Scenario 2. Hence, significant reduction of the AFPR can be obtained when farmers participate in a multi-year YP.

In addition to the feature of lower AFPRs in multi-year plans, another significant feature of the proposed plan is that farmers would obtain partial payments to cover possible losses in a specific year. If farmers have yield or revenue losses in a particular year, the partial payment in the plan would prevent such losses from bankrupting them. Lower AFPRs and partial payments are expected to help farmers reduce the negative effect resulting from agricultural risks. Different strategies for the partial payment can also be considered. For example, we assumed that farmers who received partial payments in a year can keep the payments even when the sum of the actual yield over multiple years is greater than the guaranteed yield. An alternative strategy is that farmers would have to pay back the partial payment in this scenario. Further research will be required to evaluate the AFPRs when different payment strategies are considered.

To demonstrate that our proposed insurance plans are practical, we evaluate the correlation structures for representative counties in different states by using county-level data for corn in Iowa, Illinois, Indiana, and Ohio. Based on the real data analyses, the correlation coefficients are generally small. Therefore, if the proposed multi-year insurance plans are implemented, AFPR will be lower than the rate currently seen in single year insurance plans. The autocorrelation of order 1 results in Table 3 can provide a guideline for government agencies to prioritize counties or states and crops to implement multi-year insurance plans. For example, AFPR for corn in Greene County in Ohio could be much lower based on the multi-year insurance plan than the single year plan due to the significantly negative autocorrelation.

We also demonstrate how two- and three-year YP policies can be implemented by using county-level data from Adair County in Iowa. We show that partial payments can help farmers stabilize their incomes. Our results provide strong evidence that producers can benefit from multi-year insurance plans. Policymakers should seriously consider multi-year insurance plans as a viable alternative to currently available insurance programs.

A multi-year crop insurance contract is designed to help farmers stabilize their incomes with possible lower premium cost through the diversification of yield risks over multi-year. Farmers’ preferences for the multi-year crop insurance is related to time preference and risk aversion, and risk situation (yield and price risk). When farmers prefer to obtain the claim in the early stage, they may prefer higher single-year coverage level. This is an interesting research area to incorporate time preference and risk aversion to investigate farmers’ preference for a multi-year crop insurance contract.

In our analysis, we did not consider the general equilibrium from the farmer’s perspective in terms of the utility function and the government’s perspective through the objective function. Arrow [21] has shown that an insurance model with a deductible is generally Pareto optimal. Besides, both farmers and government insurers would enter into the transactions of insurance contracts from year to year whether the contracts are single-year or multi-year. Therefore, fully modeling farmers’ and government’s behavior of a crop insurance contract might require the consideration of time profiles of pricing for government insurers and choices about whether to insure each year, and for farmers, the choices about purchasing a two single-year or a two-year crop insurance contracts. These issues are interesting areas that will be investigated further in the future.

## Supporting Information

S1 Table Parameter estimates for detrended data and goodness-of-fit test. (DOCX)

## References

[1] Gardner BL, Kramer RA (1986) Experience with Crop Insurance Programs in the United States; Hazel P, Pomareda C, Valdez A, editors. Baltimore: Johns Hopkins University Press.

[2] Chambers RG (1989) Insurability and Moral Hazard in Agricultural Insurance Markets. American Journal of Agricultural Economics 71: 604–616.

[3] Rogerson WP (1985) The 1st Order Approach to Principal-Agent Problems. Econometrica 53: 1357–1367.

[4] Kleindorfer PR, Kunreuther H, Ou-Yang C (2012) Single-year and multi-year insurance policies in a competitive market. Journal of Risk and Uncertainty 45: 51–78.

[5] Maynard T, Ranger N (2012) What Role for "Long-term Insurance" in Adaptation? An Analysis of the Prospects for and Pricing of Multi-year Insurance Contracts. Geneva Papers on Risk and Insurance-Issues and Practice 37: 318–339.

[6] Osipenko M, Shen Z, Odening M (2015) Is there a demand for multi-year crop insurance? Agricultural Finance Review 75: 92–102.

[7] Goodwin BK (2009) Payment Limitations and Acreage Decisions under Risk Aversion: A Simulation Approach. American Journal of Agricultural Economics 91: 19–41.

[8] Woodard JD, Sherrick BJ, Schnitkey GD (2010) Revenue Risk-Reduction Impacts of Crop Insurance in a Multicrop Framework. Applied Economic Perspectives and Policy 32: 472–488.

[9] Skees JR, Reed MR (1986) Rate Making for Farm-Level Crop Insurance—Implications for Adverse Selection. American Journal of Agricultural Economics 68: 653–659.

[10] Miranda MJ, Glauber JW (1997) Systemic risk, reinsurance, and the failure of crop insurance markets. American Journal of Agricultural Economics 79: 206–215.

[11] Vedenov D (2008) Application of Copulas to Estimation of Joint Crop Yield Distributions. American Agricultural Economics Association Annual Meeting. Orlando.

[12] Tejeda HA, Goodwin BK (2008) Modeling Crop prices through a Burr distribution and Analysis of Correlation between Crop Prices and Yields Using a Copula method. American Agricultural Economics Association Annual Meeting. Orlando, FL, USA.

[13] Goodwin BK, Hungerford A (2015) Copula-Based Models of Systemic Risk in US Agriculture: Implications for Crop Insurance and Reinsurance Contracts. American Journal of Agricultural Economics 97: 879–896.

[14] Okhrin O, Odening M, Xu W (2013) Systemic Weather Risk and Crop Insurance: The Case of China. Journal of Risk and Insurance 80: 351–372.

[15] Botts RR, Boles JN (1958) Use of Normal-Curve Theory in Crop Insurance Ratemaking Journal of Farm Economics 40: 733–740.

[16] Gallagher P (1987) U.S. Soybean Yields: Estimation and Forecasting with Non-Symmetric Disturbances. American Journal of Agricultural Economics 69: 798–803.

[17] Nelson CH (1990) The Influence of Distribution Assumptions on the Calculation of Crop Insurance Premia. North Central Journal of Agricultural Economics 12: 71–78.

[18] Borges RB, Thurman WN (1994) Marketing Quotas and Random Yields—Marginal Effects of Inframarginal Subsidies on Peanut Supply. American Journal of Agricultural Economics 76: 809–817.

[19] Babcock BA, Hennessy DA (1996) Input demand under yield and revenue insurance. American Journal of Agricultural Economics 78: 416–427.

[20] Coble KH, Knight TO, Pope RD, Williams JR (1996) Modeling farm-level crop insurance demand with panel data. American Journal of Agricultural Economics 78: 439–447.

[21] Arrow KJ (1985) The Economics of Agency; Pratt J, Zeckhauser R, editors. Boston: Harvard Business School Press.

