# Supplementary material for: Policy Design of Multi-Year Crop Insurance Contracts with Partial Payments
Source: PLoS One. 2015 Dec 22;10(12):e0145384. doi: 10.1371/journal.pone.0145384 (PMC4687925; doi:10.1371/journal.pone.0145384)
Supplement: S1 Table — (DOCX) [file pone.0145384.s001.docx]

**S1 Table. Parameter Estimates for detrended data and goodness-of-fit test**

| Parameter Estimate for Detrended Data | | |
| --- | --- | --- |
| Variable | Parameter estimate | P-value |
| Intercept | 28.82 | <0.0001 |
| T | 1.14 | 0.0014 |
| t^2^ | 0.006 | 0.13 |
| Goodness-of-fit Test for Normal Distribution | | |
| Test | Statistic (P-value) | |
| Kolmogorov-Smirov | 0.101 (0.04) | |
| Cramer-von Mises | 0.206 (<0.005) | |
| Anderson-Darlilng | 1.236 (<0.005) | |
| Chi-Square | 16.325 (0.003) | |
| Goodness-of-fit Test for Beta Distribution | | |
| Chi-Square | 11.337 (0.023) | |
